# Using a Passive Back Exoskeleton During a Simulated Sorting Task: Influence on Muscle Activity, Posture, and Heart Rate

**DOI:** 10.1177/00187208211073192

**Published:** 2022-02-27

**Authors:** Mona Bär, Tessy Luger, Robert Seibt, Monika A. Rieger, Benjamin Steinhilber

**Affiliations:** 155909 University of Tübingen and University Hospital Tübingen, Tübingen, Germany

**Keywords:** Assistive device, electromyography, back support, working posture, nonneutral trunk postures

## Abstract

**Objective:**

To evaluate using a back exoskeleton in a simulated sorting task in a static forward bent trunk posture on muscle activity, posture, and heart rate (HR).

**Background:**

Potentials of exoskeletons for reducing musculoskeletal demands in work tasks need to be clarified.

**Methods:**

Thirty-six healthy males performed the sorting task in 40°-forward bent static trunk posture for 90 seconds, in three trunk orientations, with and without exoskeleton. Muscle activity of the erector spinae (ES), biceps femoris (BF), trapezius descendens (TD), rectus abdominis (RA), vastus laterals (VL), and gastrocnemius medialis was recorded using surface electromyography normalized to a submaximal or maximal reference electrical activity (%RVE (reference voluntary electrical activity)/%MVE). Spine and lower limb postures were assessed by gravimetric position sensors, and HR by electrocardiography.

**Results:**

Using the exoskeleton resulted in decreased BF muscle activity [−8.12%RVE], and minor changes in ES [−1.29%MVE], RA [−0.28%RVE], VL [−0.49%RVE], and TD [+1.13%RVE] muscle activity. Hip and knee flexion increased [+8.1°; +6.7°]. Heart rate decreased by 2.1 bpm. Trunk orientation had an influence on BF muscle activity.

**Conclusion:**

Using the back exoskeleton in a short sorting task with static trunk posture mainly reduced hip extensor muscle activity and changed lower limb but not spine posture. Implications of using a back exoskeleton for workers’ musculoskeletal health need further clarification.

**Application:**

The detected changes by using the Laevo® illustrate the need for further investigation prior to practical recommendations of using exoskeletons in the field. Investigating various work scenarios in different kind of workers and long-term applications would be important elements.

## INTRODUCTION

Musculoskeletal disorders (MSD) in the back area, including low back pain (LBP), represent the most reported health problem among the working population in the European Union (DeKok et al., 2019). Low back pain has been reported to have an estimated lifetime prevalence up to 75% among the general population (Burton & Kendall, 2014) and a 12-month prevalence ranging from 25% among employees in the United States (Dick et al., 2020) to 43% among workers in the European Union (DeKok et al., 2019). The consequences include limitations in daily life of the persons concerned (Eurostat, 2010) as well as impairments at and absence days from work (DeKok et al., 2019; Valirad et al., 2015). Several physical risk factors have been reported to be related to LBP, including working in awkward positions like trunk forward flexion, heavy physical work and lifting (Coenen et al., 2014; Coenen et al., 2013; da Costa & Vieira, 2010; Dick et al., 2020).

Lately, practitioners and researchers have focused on the occupational application of exoskeletons for reducing physical workload by supporting movements and postures of workers (Bär et al., 2021; Toxiri et al., 2019). “Exoskeletons are assistive systems worn on the body that act mechanically on the body. In an occupational context, they aim to support functions of the skeletal and locomotor system during physical work.” (Steinhilber, Luger, et al., 2020). For supporting the lower back in work tasks, several passive exoskeletons have been described; some are already commercially available and others still are in a developmental stage. Most of the commercially available exoskeletons use passive components to generate an assistive torque for storing or releasing energy, such as spring-like structures or soft elastic bands (Toxiri et al., 2019).

A variety of studies evaluated passive back supporting exoskeletons (BSEs) in an occupational context regarding their effects on acute physical stress and strain. The meta-analyses of a recent systematic review indicated the capability of using BSEs to reduce muscle activity in the supported areas (Bär et al., 2021), which is frequently used as objective physical strain indicator reflecting the necessary amount of muscle activation to realize a given motor task when normalized to the muscle activity during a maximum voluntary contraction (MVC) (Burden, 2010). Most of the included studies were performed in the laboratory and evaluated dynamic tasks, like lifting and lowering. Only few studies examined BSEs in static postures including forward trunk bending (Bär et al., 2021; Madinei et al., 2020b). However, there is some evidence for reduced back extensor muscle activity ranging between 11%-61% (Agnew, 2008; Bosch et al., 2016; Graham et al., 2009; Koopman et al., 2019; Madinei et al., 2020b; Ulrey & Fathallah, 2013; Wei et al., 2020) and reduced hip extensor muscle activity ranging between 17%-24% (Bosch et al., 2016; Ulrey & Fathallah, 2013) when using a BSE in static forward bent postures. Within these studies, the reported reductions have not always reached statistical significance (Ulrey & Fathallah, 2013), and in some of the various observed bending postures there have been no changes or even increases in trunk extensor muscle activity (Koopman et al., 2019; Madinei et al., 2020b). One main function of exoskeletons is the load transfer to other body areas (Toxiri et al., 2019; Weston et al., 2018). However, physical stress and strain parameters in these non-supported areas have been evaluated rarely (Bär et al., 2021) and most of the studies have not included posture into their observations so far (Kermavnar et al., 2021).

Physical back loading might be reduced by using a passive BSE in work tasks requiring static postures holding the trunk in a forward bent position. However, from the existing literature no conclusions can be drawn yet on their effectiveness with respect to LBP reduction and prevention or on the occurrence of possible collateral effects. Furthermore, most of the existing studies on this topic only observed working postures in the sagittal plane and did not include an orientation of the trunk to the side, that is, rotation which is often required at workplaces and may modify the intended support provided by the exoskeleton due to modified individual inclination angles. Therefore, this study evaluated the effects of using a passive BSE (Laevo® V2.56) during a sorting task with 40°-trunk forward flexion, with and without additional trunk rotation induced by a 45°-sideward workstation orientation. During initial measurements, a direct increase in physiological response such as heart rate (HR) and muscle activity within the first seconds was observed and lasted on a steady state over several minutes. Thus, for detecting acute effects of the exoskeleton and also avoiding muscular fatigue, a 90-second period for performing the sorting task was chosen. We included the outcome measures muscle activity, body posture, and HR. Concerning the exoskeletons’ function, our primary outcome measure in this study was muscle activity of the erector spinae (ES) and biceps femoris (BF), which are responsible for back and hip extension. We hypothesized that both ES and BF muscle activity are reduced when wearing the exoskeleton. The secondary outcomes were muscle activity of the vastus lateralis (VL), gastrocnemius medialis (GM), trapezius descendens (TD), and rectus abdominis (RA), posture of the spine as well as hip flexoin (HF) and knee flexion (KF), and HR. We had no particular expectations with respect to the secondary outcomes. We included VL and GM because these muscles might be affected due to load shifting when wearing the exoskeleton. We included TD because the exoskeleton shoulder straps might bother wearers. We included RA because it acts antagonistic to the ES, and trunk bending might be hindered by the exoskeletons’ extension moment resulting in increased RA muscle activity. Body posture might be influenced by wearing the exoskeleton, as previous studies reported that wearers complained about reduced freedom of movement (Baltrusch et al., 2019). We included HR to monitor an eventual cardiovascular response of using the exoskeleton.

## METHODS

### Sample Size and Study Design

**Figure 1.**
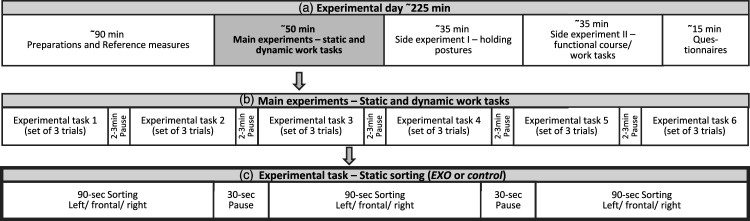
Line (a) shows one exemplary experimental day for one subject. Line (b) shows the sequence of the six main experimental conditions (randomized order): Static_sorting_*EXO*/Static_sorting_*control*/Dynamic_lifting_Stoop_posture_*EXO*/Dynamic_lifting_Stoop_posture_*control*/ Dynamic_lifting_Squat_posture_*EXO*/Dynamic_lifting_Squat posture_*control* which were performed three times each (including three *Trunk orientations*). Each set of static sorting tasks lasted 330 s, and each set of dynamic lifting tasks lasted 375 s. Line (c) shows one set of the static sorting task which is the basis of this manuscript. *Trunk orientations* (left/frontal/right) were performed in randomized order.

This manuscript describes one part of a larger, explorative laboratory study, evaluating the Laevo® V2.56 exoskeleton on physiological and biomechanical parameters (ClinicalTrials.gov, NCT03725982). The overall study was designed according to the Declaration of Helsinki and approved by the Ethics Committee of the University and University Hospital of Tübingen (617/2018BO2). The overall study required a sample size of 36 subjects based on a Single Williams Latin Square design (Luger, Bär, Seibt, Rimmele, et al., 2021). The current article focuses on the static sorting task (Figure 1), for which we maintained a within-subject design with *Device* (without (control) vs. with (EXO)) and *Trunk orientation* (ipsilateral vs. frontal vs. contralateral) as the within-subject variables. Randomization was realized by drawing three lots: (1) order of *control* and EXO; (2) order of the *Trunk orientation* (left, frontal, right); (3) measured body side reflecting muscle activity and HF and KF angles. All randomizations were balanced across the subjects.

### Participants

Thirty-six healthy males (mean age 25.9 ± 4.6 years, mean body height 178.7 ± 7.3 cm, mean body weight 73.5 ± 8.9 kg) participated in the study. Inclusion criteria were: 18–40 years of age, BMI of 18.5–30 kg/m^2^, free of any acute or cardiovascular diseases, physical disability, systemic diseases, or neurological impairments that would hinder the subject from performing the tasks and wearing the exoskeleton. Only male subjects were included due to a continuing domination of male workers in the manufacturing industries.

### Experimental Procedure and Task

On a first day lasting 1 h, the subjects got informed about the study procedure and signed the informed consent. Inclusion and exclusion criteria were clarified and anthropometric measures (i.e. body height and weight) were collected. The required exoskeleton size (S/L) was chosen and the exoskeleton was adjusted to best fit the subjects. Subsequently, subjects got familiarized with wearing the exoskeleton and performing the task. On a second day lasting 4 h, the subjects got prepared with the measurement equipment and performed the six conditions of the simulated static sorting task with either a 30 s or 120 s rest break in between (Figure 1).

**Figure 2.**
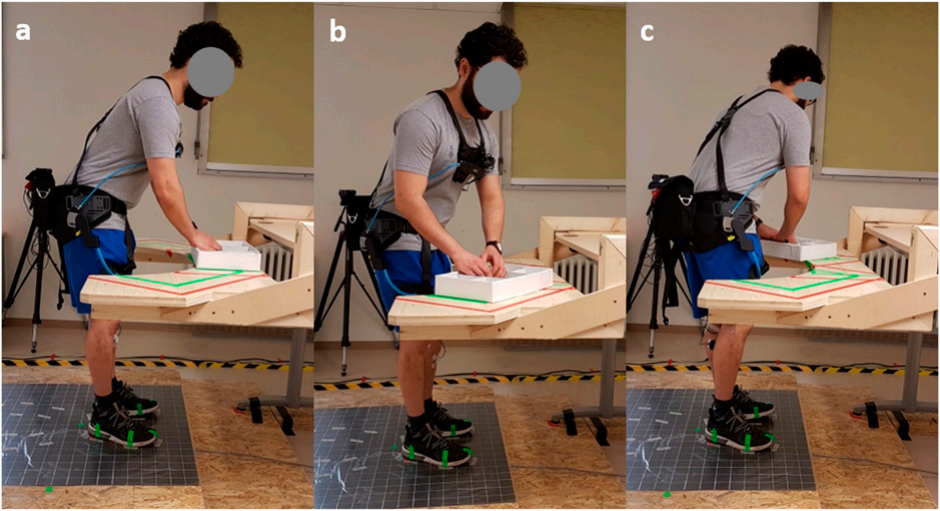
Sorting task; (a) frontal; (b) 45° trunk orientation to the right; (c) 45° trunk orientation to the left.

The task included sorting screws and pins for 90 s with the trunk bent forward in 40°. This inclination angle was ensured by the signal of a position sensor placed on the spinous process of the 10^th^ thoracic vertebrae (T10) which was visually controlled on a screen by the researchers. The mean T10 inclination angle was 38.9° (cf. Table 2). The feet position was kept constant during all experimental conditions and defined prior to the experiment and marked in the study setup, while participants were requested to stand comfortably upright, positioning their feet equably, facing straight ahead, and extending but not overstretching their legs. Trunk orientations deviating from the sagittal plane were realized by positioning the task setup in a 45°-rotation from the sagittal (Figure 2). The different feet and posture requirements were continuously controlled for by the researchers and corrected by verbal instructions when necessary.

### Exoskeleton

The Laevo® exoskeleton (V2.56, Laevo B.V., Delft, the Netherlands; 2.8 kg) is a passive BSE that supports back extension. The exoskeleton consists of a hip belt with two attached joints including a gas pressure spring located close to the pivot of the hip joints. A chest pad and two leg pads are attached to the springs over rigid metal bars, movable by two-dimensional joints in the chest pad. Bending the trunk forward compresses the springs and generates a moment which supports back and hip extension. The springs can be turned off and be adjusted to start the support at different trunk flexion angles (ranging from 0° to 45° in steps of 5°). Depending on the subject’s body proportions, the support angle was adjusted to assure contact but no pressure on the chest by the chest pad during an upright standing position (i.e., 0Nm), monitored by a force sensor built into the chest pad (38×10 mm; Type KM38-1 kN, ME-Messsysteme GmbH, Henningsdorf, Germany) and visually controlled on a screen by the researchers.

## Measurement and Data Analysis

### Muscle activity

The muscle activity of erector spinae lumbalis at level of lumbar vertebrae L1 (ES), BF, RA, VL, GM and TD muscles was recorded unilaterally (body side was randomized) via surface electromyography (EMG). This procedure was due to practical reasons of having no more EMG channels left. The skin over the muscles was shaved and cleaned using abrasive paste (Skin Prep Gel, Nuprep®, Aurora, USA). Pre-gelled Ag/AgCl surface electrodes with an active area of 15 mm diameter (KendallTM H93SG electrocardiography (ECG) Electrodes, Covidien, Zaltbommel, the Netherlands) were used in bipolar configuration (inter-electrode center distance 25 mm) and located over the muscle bellies according to international standards (Criswell, 2010; Hermens et al., 2000). The electrodes over the VL were in some cases placed slightly more distal compared to the standard recommendations to avoid any contact with the exoskeleton’s leg pad. The ground electrode was placed over cervical vertebrae C7 (PS12-II, THUMEDI® GmbH & Co. KG, Thum, Germany, physical resolution 24 bit; overall CMRR >98 dB; overall effective sum of noise <0.5 μV RMS; linearity typically ±0.1 dB at 30−1200 Hz). Electromyography raw signals were differential amplified, filtered (high-pass, second order, −3 dB at 4 Hz; low-pass, 11th order, −3 dB at 1300 Hz), sampled (4096 Hz), analog-digital-converted, analyzed, and continuously stored. The signals were real-time transformed in the frequency domain (1024-point Fast Fourier Transformation using a Bartlett-window with 50% overlap), digitally filtered (high-pass, 11^th^ order, −3 dB at 16 Hz) and powerline interferences were removed by an average filter (11th order, −3dBat 50 Hz and its first seven harmonics, bandwidth of 4 Hz was replaced by its spectral neighbors). The electrical activity (eA) [μV] was calculated as the root-mean-square (RMS) of the EMG amplitude by real-time estimation (250 ms moving window with 50% overlap) from the power spectrum and stored synchronously to the raw data.

The eA of the BF, VL, RA, GM, and TD were normalized to the eA of a submaximal reference voluntary contraction (Supplemental Appendix 1), presented as reference voluntary eA (%RVE; Mathiassen et al., 1995). RVE normalization was done since less demanding than MVC based normalization and less affected by motivational aspects (Steinhilber & Rieger, 2013). The eA of the ES, was normalized using a maximal voluntary eA (%MVE; Mathiassen et al., 1995) during MVC, since this evaluated muscle is located in the targeted area of the exoskeleton and has often been reported expressed as %MVE in studies already investigating the Laevo® exoskeleton (Bosch et al., 2016; Koopman et al., 2019). The median (50th percentile) normalized muscle activity [%MVE/%RVE] was calculated for each experimental condition: *control* and *EXO*, *ipsilateral* (measured side equals trunk orientation), *frontal*, and *contralateral* (measured side opposes trunk orientation) over the 90-second task.

### Body posture

For recording body posture, we used two-dimensional gravimetric position sensors (PS12-II; Thumedi GmbH & Co. KG) placed on the skin over thoracic vertebrae T1 and T10, lumbar vertebrae L1 and L5, and on the anterior side of the femur and tibia, fixed with double-sided adhesive tape (25×5 mm, 3M transparent Medical Standard, Top Secret®, Gesellschaft für Haarästhetik mbH, Fürth, Germany). The sensors continuously measured inclination angles respective to the gravitational axis in anteroposterior direction (resolution 0.1° and 125 ms in time; maximum static error 0.5°; maximum repetition error 0.2°). For further evaluations the differences between angles during the experimental conditions and a reference posture were used (Supplemental Appendix 1); subtracting the reference angles from the experimental angles. Four joint angles were calculated: thoracic kyphosis (TK), lumbar lordosis (LL), hip flexion (HF), and knee flexion (KF) (Table 1; Supplemental Appendix 2). The medians were calculated for each condition over the 90-second tasks. HF and KF were grouped into *ipsilateral*, *frontal,* and *contralateral*. TK and LL were grouped into *frontal* and *lateral* (including both, left and right trunk orientations).

**TABLE 1:** Calculation and Interpretation of the four joint angles

Joint angle	Calculation	Interpretation
Thoracic kyphosis	Anteroposterior inclination angles or tangent lines (Cobb method) over the processus spinosi of T1 and L1 according to the Cobb angle boundaries (Takács et al., 2018)	0° reflects curvature in upright stance posture, a positive value means the upper back is curving more (direction kyphosis), and negative value means the upper back is curving less (direction lordosis) compared to upright stance
Lumbar lordosis	Anteroposterior inclination angles or tangent lines (Cobb method) over the processus spinosi of L1 and L5 according to the Cobb angle boundaries (Takács et al., 2018)	0° reflects curvature in upright stance, a positive value means the lower back is curving less (direction kyphosis), a negative value means the lower back is curving more (direction lordosis) compared to upright stance
Hip flexion	Difference value between the anteroposterior inclination angles of L5 and femur	Smaller angles [°] reflect more hip extension (direction upright stance) and greater angles reflect more hip flexion
Knee flexion	Difference value between the anteroposterior inclination angles of femur and tibia	Smaller angles [°] reflect more knee extension (direction upright stance) and greater angles reflect more knee flexion

### Heart rate

The HR was recorded continuously using ECG sampled at 1,000 Hz (PS12-II; Thumedi GmbH & Co.KG). Two pre-gelled Ag/AgCl surface electrodes with an active area of 15 mm diameter (KendallTM H93SG ECG Electrodes, Covidien, Zaltbommel, the Netherlands) were placed ∼5 cm cranial and ∼3 cm lateral from the distal end of the sternum and over the anterior to mid-axillary line at the fifth left rib. The median HR was calculated over the 90-second periods for the conditions *frontal* and *lateral*.

### Statistical Analysis

We visually inspected the data histograms including their skewness and kurtosis. Electromyography and position sensor data showed no normal distribution. Subsequently, a log-transformation (LOG10) for the statistical analysis was performed. Heart rate data showed normal distribution, which was used directly for further evaluation. Differences between conditions were analyzed by repeated-measures analyses of variance with the fixed factors *Device* (*EXO* vs. *control*) and *Device × Trunk orientation* (three levels: *ipsilateral* vs. *frontal* vs. *contralateral*, for the outcome parameters muscle activity, KF and HF; or two factors: *frontal* vs. *lateral*, for the outcome parameters TK, LL and HR). In case of significant interaction effects, we used Tukey HSD for post hoc pairwise comparisons. We calculated F-values, *p*-values and effect size partial eta squared $\left({\text{η}}_{\text{p}}^{2}\right)$ using the F-ratios strategy (Bakeman, 2005) for fixed effects, and T-value, *p*-value and effect size Cohen’s d using the pooled standard deviation strategy (Cohen, 1988) for post hoc pairwise comparison. Effect sizes are interpreted by Cohen (1988) as follows: small ( ${\text{η}}_{\text{p}}^{2}$ ≥0.02; d≥0.2), medium ( ${\text{η}}_{\text{p}}^{2}$ ≥0.13; d≥0.5), or large ( ${\text{η}}_{\text{p}}^{2}$ ≥0.26; d≥0.8). A significance level of α≤0.05 was used. All statistical evaluations were performed in JMP® (Version 14.2.0, SAS Inc., Carry, NC, USA).

## RESULTS

Descriptive information about the trunk inclination angle and the supporting moment of the exoskeleton are provided in Table 2.

**TABLE 2:** Descriptive Information (mean ± SD) of the trunk inclination angle as measured at vertebra T10 and supporting moment provided by the exoskeleton as measured by the force sensor in the exoskeleton’s chest pad

	Trunk orientation	Control	Exoskeleton
**Trunk inclination [°]**	Left	37.97 (±3.72)	38.63 (±3.86)
	Frontal	39.42 (±3.65)	39.92 (±4.12)
	Right	38.39 (±3.72)	38.90 (±3.86)
**Supporting moment [Nm]**	Ipsilateral	-	23.25 (±5.77)
	Frontal	-	22.81 (3.75)
	Contralateral	-	22.67 (±4.20)

Median values with interquartile ranges (IQR) or mean values with standard deviations (SD), and differences between the exoskeleton conditions are provided in Table 3a for muscle activity, 3b for posture and 3c for HR.

**TABLE 3:** Median or mean values, corresponding IQR or SD, absolute and relative differences showing *EXO* compared to c*ontrol*. (a) Median RMS of normalized eA (b) Median angles (c) Mean heart rate.

a		Control		EXO		Difference	
Muscle activity [%MVE/%RVE]	Trunk orientation	Median	(IQR)	Median	(IQR)	[%MVE/%RVE]	[%]
**Erector spinae [%MVE]**	Total	12.25	(6.87)	10.96	(5.58)	−1.29	−10.5
	Ipsilateral	10.44	(5.93)	9.97	(4.83)	−0.47	−4.5
	Frontal	12.74	(7.02)	11.55	(6.70)	−1.19	−9.4
	Contralateral	13.60	(6.95)	11.28	(6.24)	−2.32	−17.1
**Biceps femoris [%RVE]**	Total	35.98	(28.99)	27.86	(25.09)	−8.12	−22.6
	Ipsilateral	32.93	(22.58)	20.28	(22.78)	−12.65	−38.4
	Frontal	35.72	(26.98)	30.40	(22.18)	−5.32	−14.9
	Contralateral	39.63	(33.06)	35.75	(33.2)	−3.88	−9.8
**Rectus abdominis [%RVE]**	Total	1.85	(1.74)	1.57	(1.38)	−0.28	−15.2
	Ipsilateral	1.90	(1.66)	1.57	(1.55)	−0.33	−17.3
	Frontal	1.83	(1.66)	1.54	(1.66)	−0.29	−15.8
	Contralateral	1.82	(1.90)	1.71	(1.37)	−0.12	−6.5
**Vastus lateralis [%RVE]**	Total	3.85	(3.58)	3.35	(2.72)	−0.49	−12.8
	Ipsilateral	4.58	(15.75)	3.47	(6.22)	−1.1	−24.1
	Frontal	3.88	(3.51)	3.23	(1.65)	−0.65	−16.7
	Contralateral	3.34	(2.32)	3.18	(2.84)	−0.15	−4.6
**Gastrocnemius medialis [%RVE]**	Total	54.11	(46.96)	54.11	(38.73)	+0.67	+1.2
	Ipsilateral	56.77	(46.78)	50.33	(48.27)	−6.45	−11.4
	Frontal	68.93	(29.27)	66.39	(37.09)	−2.54	−3.7
	Contralateral	25.05	(24.75)	44.49	(32.82)	+19.44	+77.6
**Trapezius descendens [%RVE]**	Total	3.77	(6.12)	4.90	(7.39)	+1.13	+30.1
	Ipsilateral	4.36	(4.21)	4.94	(6.37)	+0.59	+13.5
	Frontal	4.72	(7.7)	4.93	(11.69)	+0.21	+4.4
	Contralateral	3.45	(5.57)	4.71	(7.41)	+1.26	+36.4

b		Control		EXO		Difference	
Posture [°]	Trunk orientation	Median	(IQR)	Median	(IQR)	[°]	[%]
**Thoracic kyphosis**	Total	14.53	(12.64)	12.45	(9.74)	−2.08	−14.3
	Frontal	11.35	(12.25)	10.60	(10.36)	−0.75	−6.6
	Lateral	15.63	(10.95)	13.08	(10.00)	−2.55	−16.3
**Lumbar lordosis**	Total	12.30	(5.64)	12.38	(4.86)	+0.07	+0.6
	Frontal	13.60	(5.14)	12.85	(6.28)	−0.75	−5.5
	Lateral	12.18	(5.83)	11.95	(4.93)	−0.23	−1.8
**Hip flexion**	Total	31.93	(14.59)	40.00	(13.16)	+8.08	+25.3
	Ipsilateral	33.23	(15.76)	39.95	(13.75)	+6.73	+20.2
	Frontal	33.00	(15.20)	40.65	(12.64)	+7.65	+23.2
	Contralateral	26.25	(14.73)	36.58	(14.90)	+10.33	+39.3
**Knee flexion**	Total	10.73	(13.36)	17.45	(16.33)	+6.73	+62.7
	Ipsilateral	11.28	(13.03)	17.48	(13.45)	+6.20	+55.0
	Frontal	10.53	(11.85)	16.83	(17.14)	+6.30	+59.9
	Contralateral	10.10	(14.26)	19.70	(17.28)	+9.60	+95.0

c		Control		EXO		Difference	
Heart rate [bpm]	Trunk orientation	Mean	*(SD)*	Mean	*(SD)*	[bpm]	[%]
	Total	85.29	(10.19)	83.16	(10.49)	−2.13	−2.5
	Frontal	85.58	(10.37)	83.25	(10.73)	−2.33	−2.7
	Lateral	85.14	(10.17)	83.11	(10.44)	−2.03	−2.4

IQR = interquartile range; SD = standard deviation; RMS = root mean square; eA = electrical activity; MVE/RVE = maximal/reference voluntary electrical activity; bpm = beats per minute; ipsilateral: the measured side equaling the working direction; contralateral: the measured side opposing the working direction; lateral: includes both sideward working directions.

Corresponding statistics for main effects of the exoskeleton condition *Device* (*EXO* vs. *control),* the interaction effects between *Device* and *Trunk orientation* (*Device × Trunk orientation*) and the pairwise comparisons for *Device × ipsilateral*, *Device × frontal,* and *Device × contralateral* or *Device × lateral* are provided in Table 4a for muscle activity, 4b for posture and 4c for HR.

**TABLE 4:** F-values and *p*-values of the repeated measures ANOVAs with corresponding effect sizes (partial eta squared or Cohens´ d (*d*)). Main effects of the exoskeleton condition (*Device*) and the interaction effects for device with trunk orientation (*Device × Trunk orientation*). Pairwise comparison for variables with significant interaction effects. (a) log-transformed RMS values of the normalized eA of the muscles. (b) log-transformed angles (c) HR.

a	Main effect			Interaction effect			Pairwise comparisons of *EXO* vs. *Control* in three *Trunk orientations*					
Muscle activity	Device			Device *×* Trunk orientation			Ipsilateral		Frontal		Contralateral	
	*F*	*p*	${\text{η}}_{\text{p}}^{2}$	*F*	*p*	${\text{η}}_{\text{p}}^{2}$	*p*	*d*	*p*	*d*	*p*	*d*
**Erector spinae**	8.15	0.007*	**0.193** ^**μ**^	0.82	0.446	0.023^**σ**^	-	-	-	-	-	-
**Biceps femoris**	22.26	<0.001*	**0.389** ^**λ**^	3.27	0.044*	**0.085** ^**σ**^	<0.001*	**−0.414** ^**σ**^	0.021*	**0.359**^**σ**^	0.732	−0.280^**σ**^
**Rectus abdominis**	28.67	<0.001*	**0.450** ^**λ**^	1.42	0.248	0.039^**σ**^	-	-	-	-	-	-
**Vastus lateralis**	6.19	0.018*	**0.155** ^**μ**^	2.22	0.117	0.063^**σ**^	-	-	-	-	-	-
**Gastrocnemius medialis**	0.33	0.57	0.009	5.97	0.004*	**0.146** ^**μ**^	0.810	−0.051	1	−0.075	0.090	0.109
**Trapezius descendens**	10.75	0.002*	**0.235** ^**μ**^	0.78	0.460	0.022^**σ**^	-	-	-	-	-	-

b	Main effect			Interaction effect			Pairwise comparisons of *EXO* vs. *Control* in two/three *Trunk orientations*					
Posture	Device			Device *×* Trunk orientation			Lateral/Ipsilateral		Frontal		Contralateral	
	*F*	*p*	${\text{η}}_{\text{p}}^{2}$	*F*	*p*	${\text{η}}_{\text{p}}^{2}$	*p*	*d*	*P*	*d*	*p*	*d*
**Thoracic kyphosis**	1.27	0.268	0.038^**σ**^	0.69	0.411	0.021^**σ**^	-	-	-	-	n/a	n/a
**Lumbar lordosis**	0.47	0.499	0.012	1.87	0.180	0.048^**σ**^	-	-	-	-	n/a	n/a
**Hip flexion**	38.72	<0.001*	**0.525** ^**λ**^	3.22	0.046*	**0.084** ^**σ**^	<0.001*	**0.839** ^**λ**^	<0.001*	**0.917** ^**λ**^	<0.001*	**0.876**^**λ**^
**Knee flexion**	34.39	<0.001*	**0.496** ^**λ**^	2.76	0.071	0.073^**σ**^	-	-	-	-	-	-

c	Main effect			Interaction effect			Pairwise comparisons of *EXO* vs. *Control* in two *Trunk orientations*					
**Heart rate**	Device			Device *×* Trunk orientation			Lateral		Frontal		
	*F*	*p*	${\text{η}}_{\text{p}}^{2}$	*F*	*p*	${\text{η}}_{\text{p}}^{2}$	*p*	*d*	*p*	*d*		
20.13	<0.001*	**0.339** ^**λ**^	0.22	0.643	0.006	-	-	-	-		

RMS = root mean square; eA = electrical activity.

*Significant *p*-value (α≤0.05); ^**λ**^ large effect size ( ${\text{η}}_{\text{p}}^{2}$ ≥0.26/d≥0.8); ^**μ**^ medium effect size ( ${\text{η}}_{\text{p}}^{2}$ ≥0.13/d≥0.5); ^**σ**^ small effect size ( ${\text{η}}_{\text{p}}^{2}$ ≥0.02/d≥0.2).

### Muscle Activity

A significant main effect of *Device* for ES occurred when using the *EXO* resulting in a decreased muscle activity (−1.3%MVE; *p* = 0.007; ${\text{η}}_{\text{p}}^{2}$ = 0.193) without significant interaction effect for *Device × Trunk orientation*. BF had a significant main effect of *Device*; its muscle activity decreased when using the *EXO* (−8.1%RVE; *p* < 0.001; ${\text{η}}_{\text{p}}^{2}$ = 0.389). BF also showed a significant *Device × Trunk orientation* interaction effect. Its muscle activity decreased when using the *EXO* with *ipsilateral* and *frontal* orientations. RA had a significant main effect of *Device* when using the *EXO* showing a decreased muscle activity (−0.3%RVE; *p* < 0.001; ${\text{η}}_{\text{p}}^{2}$ = 0.450) and the VL had a significant main effect for *Device* when using the *EXO* showing a decreased muscle activity (−0.49%RVE; *p* = 0.018; ${\text{η}}_{\text{p}}^{2}$ = 0.155), both without significant interaction effects for *Device × Trunk orientation.* The GM had no main effect for *Device*. However, there was a significant interaction effect for *Device × Trunk orientation* without significant effects within the essential pairwise comparisons. The TD muscle had a significant main effect for *Device* showing an increased muscle activity when using the *EXO* (+1.1%RVE; *p* = 0.002; ${\text{η}}_{\text{p}}^{2}$ = 0.235) without a significant interaction effect for *Device × Trunk orientation*.

### Body Posture

*Device* had no significant main effects on TK and LL. *Device* had a significant main effect on HF, showing increased flexion angles (i.e. flexing more) when using *EXO* (+8.1°; *p* < 0.001; ${\text{η}}_{\text{p}}^{2}$ = 0.525). *Device × trunk orientation* had a significant effect on HF, but without a relation to differences between *EXO* and *Control*, meaning that the HF increased when using the *EXO,* no matter which *Trunk orientation* was applied with similar effect sizes. *Device* had a significant main effect on KF, which increased (i.e. flexing more) when using the *EXO* (+6.7°; *p* < 0.001; ${\text{η}}_{\text{p}}^{2}$ = 0.496), without a significant interaction effect for *Device × Trunk orientation.*

### Heart Rate

*Device* had a significant main effect on HR, which decreased when using the *EXO* (−2.1bpm; *p* < 0.001; ${\text{η}}_{\text{p}}^{2}$ = 0.339) without interaction effects for *Device × Trunk orientation*.

## DISCUSSION

The results of this study confirmed our hypothesis, because ES and BF muscle activity both reduced when wearing the exoskeleton. Since BF was reduced more than ES, this indicates that the exoskeleton supports hip extension to a larger extend than back extension during work tasks performed in a static forward bent trunk posture. Extra support for the latter indication is the finding that both HF and KF increased when wearing the exoskeleton. With respect to the other secondary outcomes, RA, VL, and TD muscle activity slightly changed when wearing the exoskeleton, but for GM muscle activity and spinal posture we could not detect statistically significant changes. HR slightly decreased when using the exoskeleton but no substantial effect on cardiovascular strain can be deduced from this finding. The factor trunk orientation had a significant effect on BF, so that BF muscle activity significantly reduced in the ipsilateral and frontal but not in the contralateral working direction when wearing an exoskeleton.

### Muscle Activity

The Laevo® is designed to support the lower back by providing an extension moment in, i.e., static holding tasks, aiming to reduce lower back physical loading and eventually long-term lower back complaints. Previous studies investigating work tasks requiring static ∼40° forward bent working postures presented promising results with lower back muscle activity reductions ranging from ∼8% during fastening (i.e. ∼1.5%RVE; (Luger, Bär, Seibt, Rieger, & Steinhilber, 2021)) to ∼36% during assembling (i.e. ∼3.5%MVE; (Bosch et al., 2016)). Also, the current study showed that ES muscle activity decreased by ∼11% (i.e. ∼1.5%RVE) when wearing the Laevo®. The divergent results may be the result of the tasks that were not exactly the same. When contrasting these results with studies that examined repetitive lifting and lowering, reductions in back extensor activity are slightly higher with 3–7%MVE (i.e. 8–20% relative reduction; (Baltrusch et al., 2020; von Glinski et al., 2019; Yin et al., 2019)). Although work tasks requiring static postures belong to the first factors for back MSD (da Costa & Vieira, 2010), the type of task performed may be indicative of the BSE’s efficacy, which may be stronger in lifting and lowering.

While the reduction of lower back muscular load was not as notable as desired, the muscular load on the hip extensor decreased to a greater extend with ∼23% (i.e., ∼8.1%RVE). Previous studies support this finding with reduced hip extensor muscle activities ranging from 20-36% (Bosch et al., 2016; Luger, Bär, Seibt, Rieger, & Steinhilber, 2021). This difference in muscle load reduction of the trunk and hip extensors may indicate the supportive character of the Laevo® for extending the hips because the BSE creates a hip extensor moment rather than a trunk extensor moment (Luger, Bär, Seibt, Rimmele, et al., 2021; Ulrey & Fathallah, 2013). This finding can be supported by the detected medium effect sizes for the ES and large effect sizes for the BF; however, the quantity of a muscle activity reductions which are clinically relevant is not known and therefore an interpretation in terms of preventing MSD is difficult.

Several studies observed an increased abdominal muscular load as a compensation strategy of the BSE to increase the stiffness and stabilization of the trunk (Alemi et al., 2019; Madinei et al., 2020b). The current study, however, observed a minimally reduced muscle activity level of the RA when wearing the Laevo®, although the magnitude was only 0.3%RVE. As reported by other studies, the overall activity level of the abdominal muscles is low to very low (Koopman et al., 2019; Luger, Bär, Seibt, Rieger, & Steinhilber, 2021). Therefore, the relevance of this finding with respect to consequences for musculoskeletal health may be limited. A similar interpretation may hold for the leg and shoulder muscles. Concerns were raised that muscular load may increase in the shoulder due to the exoskeleton designs (i.e. shoulder straps) and in the legs due to a load shift resulting from the changed working postures and movements due to wearing the exoskeleton (Bosch et al., 2016; Kim et al., 2020; Theurel & Desbrosses, 2019). However, the current study cannot support these theories, since marginal or no differences were found for the VL (0.5%RVE), GM (0.0%RVE) and TD (1.1%RVE; (Kim et al., 2020)). Combining these findings with the absence of arising perceived discomfort in the shoulders (Steinhilber, Bär, et al., 2020) and legs (Bosch et al., 2016) indicates that the exoskeleton may not have notable bothersome side effects.

### Body Posture

Spine posture, especially lumbar flexion, has been related to the musculoskeletal health of the low back area (Adams & Hutton, 1985). Smaller lumbar spine flexion angles tend to reduce the risk of low back disorders due to lower compression forces on the anteroposterior portions of the vertebral discs as mentioned by Ulrey and Fathallah (2013). In this study, using the *EXO* did not result in substantial spine posture changes, which is in line with two previous studies evaluating the Laevo® (Kim et al., 2020; Koopman et al., 2019). Therefore, using the Laevo® may probably not negatively influence the spine posture in work tasks requiring static forward bending trunk postures. Using other BSEs in static bending postures resulted in reduced lumbar flexion (Koopman et al., 2020; Ulrey & Fathallah, 2013) which could have beneficial effects; however, this needs further exploration.

One main function of the Laevo® exoskeleton is the load transfer to the leg and chest pads inducing pressure to these areas (Bosch et al., 2016); the resulting forces depend on the exoskeletons’ pivot point inclination angle (Koopman et al., 2019). In this study HF and KF increased when using the *EXO.* Similarly, in a fastening work task requiring a similar trunk forward bent posture (∼40°) using the *EXO* resulted in increased HF and KF (Luger, Bär, Seibt, Rieger, & Steinhilber, 2021). In comparison; using the Laevo® resulted in reduced HF in deeper trunk bending angles and systematically increased knee extension in several bending angles (Koopman et al., 2019) or resulted in knee overextension holding a likewise trunk bending angle (Bosch et al., 2016), while using another BSE holding a stooped posture with different weights carried did not result in significant HF and KF changes (Ulrey & Fathallah, 2013). Changes in lower limb posture may be a result of adjusting a comfortable and postural stable posture while performing the particular work task. The various effects on lower limb posture between studies may be caused by differences in support characteristics of the device, but also by variation in task execution (Luger, Bär, Seibt, Rieger, & Steinhilber, 2021). In this study, we explicitly prevented knee overextension by verbal instructions and visual control, wherefore lower limb posture might have been influenced. However, the most similar study results (i.e. increased HF and KF) described by Luger, Bär, Seibt, Rieger, and Steinhilber (2021) occurred when not controlling the lower limb posture as strict as in this investigation. Therefore, it is likely that the increased flexions are not provoked by our strict instructions. Using the *EXO* had a large effect on HF and KF in this investigation; however, the clinical relevance cannot be concluded from changed flexion angles alone, further parameters like joint forces should be monitored therefore in future studies.

It is indicated that using BSEs results in variable postural changes, but generally posture has not been evaluated sufficiently in previous studies. Koopman et al. (2019) showed that only very slight changes in lumbar postures may cause major changes in back muscle activity, which has been most frequently used as indicator for lower back strain in evaluations of exoskeletons (Bär et al., 2021; de Looze et al., 2016), but mainly without additionally monitoring postures. Furthermore, changes in HF angles showed significant interactions with lumbar spine moments (Koopman et al., 2019), which is an indicator for mechanical loading that has been described as a risk factor for LBP (Coenen et al., 2013). From our results, we cannot draw any conclusions of the occurring postural changes being beneficial or disadvantageous in terms of musculoskeletal loading; however, these changes illustrate the need of further investigation in terms of posture and related changes in physical load acting on the musculoskeletal system.

### Heart Rate

The slight HR reductions observed in this study (−2 bpm) when using the *EXO* do not seem to be relevant in terms of cardiovascular health, because the average HR ranged 83−85 bpm across the experimental conditions, a range that is far from the endurance limit of physical exertion (105−110 bpm) for physical work shifts (Sammito et al., 2016). Further, the here presented work task may be of too short duration for detecting changes with respect to cardiovascular strain over a complete working shift. Additionally, using a BSE in functional tasks did not significantly influence HR (Luger, Bär, Seibt, Rieger, & Steinhilber, 2021) or in longer lasting simulated working protocols decreased HR without reaching statistical significance (Godwin et al., 2009; Lotz et al., 2009). With the current knowledge we cannot state whether using a BSE has an influence, either positive or negative, on cardiovascular health.

### Interaction Effect of Device × Trunk Orientation

Symmetric postures are often hard to realize in various industries, e.g. in bricklaying (Vink & Koningsveld, 1990), and the asymmetric counterpart is associated with an increased risk for developing back disorders (Punnett et al., 1991). This was indicative for investigating the BSE not only in the traditional sagittal plane requiring a symmetric working posture (i.e. frontal trunk orientation) but also in the asymmetric counterparts, trunk rotation to the left and right. An additional reason to investigate *Trunk orientation* with respect to the efficacy of the BSE was that the BSE’s support characteristics may be dependent on the interaction between the exoskeleton’s or trunk inclination angle and the trunk orientation or rotation. With respect to the primary outcomes, we only found that *Trunk orientation* had a significant interaction effect on hip extensor muscle activity. The post hoc analyses revealed that using the exoskeleton lead to small but statistically significant interaction effects only for the *frontal* and *ipsilateral Trunk orientations* with the most prominent reductions in BF muscle activity in *ipsilateral Trunk orientation*. With respect to the secondary outcomes, a statistically significant interaction with *Trunk orientation* was found for the HF angle showing large effect sizes. Hip flexion increased in all three *Trunk orientations* but most prominent increases in the angle were in the contralateral *Trunk orientation*. Combining these two results does not provide an explanation why the BSE led to more pronounced changes in the one than in the other *Trunk orientation*. However, when comparing the three *Trunk orientations* while wearing the BSE, it seems as if a larger HF angle may result in a lower hip extension muscular load (cf. Table 3). Hip flexion when using the exoskeleton seems to depend on *Trunk orientation* and may be influenced by the amount of support provided by the exoskeleton as has been set by the “smart joint.”

### Limitations

We have to acknowledge a few study limitations. First, we included a healthy, male study population, aged 18−40 years. This does not reflect the general working population and the effects of using an exoskeleton might differ for groups of workers including all sexes (Kim et al., 2020; Madinei et al., 2020a), including healthy, symptomatic and reintegrating workers, and including aging workers. Second, the Laevo® was only adjustable to a restricted extend, where we for example experienced difficulties in placing the leg pads according to the manufacturer’s instructions (i.e., on the upper part of the thighs) in a few smaller subjects. Only two out of the 36 subjects used the S-sized exoskeleton. Further, the exoskeletons structures cannot fully be prevented from shifting when the wearer is moving; however, in this static work task we did not observe any crucial movement of the device. For avoiding a collision with the leg pad the VL electrode was placed more distal on the muscle bellies than recommended. Although the absolute muscle activation level for this muscle might possibly be less representative for the task in single subjects, this does not influence any of our outcomes as comparisons are based on a within subject design. Third, although we used a familiarization session for using the exoskeleton and executing the sorting task on a separate day, a single practice lasting 1 h may not be extensive enough for getting accustomed to the device sufficiently. According to Moyon et al. (2019) subjects reached a familiarization level four out of seven. Fourth, this experiment included a short sorting task, which does not enable to providing data on muscular fatigue or cumulative strains. Conclusions regarding the long-term use of the BSE in full work-shifts and in various tasks are thus not possible. Fifth, the trunk inclination angle was controlled for and subjects were instructed not to overstretch their knees. Consequently, possible changes in body posture may have been masked in comparison to a freestyle task execution.

## CONCLUSION

Using the Laevo® exoskeleton in a short-cyclic assembly task requiring a static forward bent posture did not result in substantial changes of lower back muscle activity, but rather reduced hip extensor muscle activity, which indicated the supportive character of hip extension. We detected increased HF and KF angles but only minor changes in spine posture. *Trunk orientation* had an impact on hip extensor muscle activity and HF angles. However, it remains unclear whether the small effects of using the Laevo® on lower back muscle activity and spinal posture and the small interacting effects of *Trunk orientation* on the hip area might have an impact on musculoskeletal health. Thus, it remains questionable if the exoskeleton has the potential to operate as intervention for back MSDs. The occurrence of changes in posture and physical strain, as well as the inconsistency between several studies indicate that further investigation on this topic is necessary before an application of exoskeletons at work can be recommended. Therefore, we suggest including several work tasks and postures, realistic working scenarios with longer lasting protocols (e.g., work shifts at least), various interrelated parameters (e.g., postures, muscle activity, moments and forces), and conducting long-term studies with populations including all genders and both healthy and symptomatic workers.

## Supplemental Material

sj-pdf-1-hfs-10.1177_00187208211073192 - Supplemental Material for Using a Passive Back Exoskeleton During a Simulated Sorting Task: Influence on Muscle Activity, Posture, and Heart Rate Click here for additional data file. Supplemental Material, sj-pdf-1-hfs-10.1177_00187208211073192 for Using a Passive Back Exoskeleton During a Simulated Sorting Task: Influence on Muscle Activity, Posture, and Heart Rate by Mona Bär, Tessy Luger, Robert Seibt, Monika A. Rieger, and Benjamin Steinhilber by Human Factors

## KEY POINTS

- The Laevo® reduced both back and hip extensor muscle activity when working in a 40°-static trunk forward bent posture; however, hip extensor muscle activity reduced to a larger extent.
- Hip and knee flexion increased when using the Laevo®, whereas spine posture remained unchanged.
- There have not been many studies evaluating the body posture in work tasks requiring a static forward bent posture, a clear conclusion about the effects of the Laevo® on working posture therefore is difficult.
- Trunk rotation, that is, task orientation, should be considered as a potential influencing factor for the impact of using the Laevo® on muscle activity and postural angles; hip extensor muscle activity as well as hip flexion was influenced by trunk orientation in this evaluation.
- The appearing changes in muscle activity, posture and heart rate cannot be interpreted to be positive or negative in terms of musculoskeletal health; they rather indicate the need of further investigation including different work tasks and longer protocols.
