# Supplementary material for: Using a Passive Back Exoskeleton During a Simulated Sorting Task: Influence on Muscle Activity, Posture, and Heart Rate
Source: Hum Factors. 2022 Feb 27;66(1):40–55. doi: 10.1177/00187208211073192 (PMC10756017; doi:10.1177/00187208211073192)
Supplement: sj-pdf-1-hfs-10.1177_00187208211073192 - Supplemental Material for Using a Passive Back Exoskeleton During a Simulated Sorting Task: Influence on Muscle Activity, Posture, and Heart Rate [file sj-pdf-1-hfs-10.1177_00187208211073192.pdf]

Appendix 1

Appendix 1. **A:** Reference contractions for the normalization procedures for the six muscles of which muscle activity was recorded using bipolar surface electromyography. **B:** Reference posture for the position sensors.

**A**	
**Muscle**	**Reference contractions**
Erector spinae	Subjects lay prone with the upper body and hips (hip bones) off the bench and the legs fixed with straps, performing *maximal* hip extension against a barrier while keeping the body horizontal and the arms crossed in front of the chest (modified Biering-Sørensen test; Biering-Sørensen, 1984). The signals from the most stable 1-second period out of the 5-seconds lasting period were used.
Biceps femoris	Subjects lay prone with 90° knee flexion, feet flexed, keeping the position while a rope with a 7-kg weight hanging over a pulley and pulling in caudal direction was attached around the ankle. The signals from the most stable 5-seconds out of the 10-seconds lasting period were used.
Rectus abdominis	Subjects lay supine with the upper body and hips off the bench and the legs fixed with straps, performing 45° hip flexion while holding an additional 10-kg weight and keeping the arms crossed in front of the chest (reverse Biering-Sørensen test; Biering-Sørensen, 1984). The signals from the most stable 5-seconds out of the 10-seconds lasting period were used.
Vastus lateralis	Subjects lay prone with 90° knee flexion, feet flexed, keeping the position while a rope with a 10-kg weight hanging over a pulley and pulling in cranial direction was attached around the ankle. The signals from the most stable 5-seconds out of the 10-seconds lasting period were used.
Gastrocnemius medialis	Subject stood upright, performing bilateral, isometric plantar flexion, raising their heels. The signals from the most stable 5-seconds out of the 10-seconds lasting period were used.
Trapezius descendens	Subject stood upright, feet hip-width apart, arms in 90° abduction but slightly in the frontal plane, elbows almost extended but not overstretched, while holding a 2-kg weight in each hand (Mathiassen et al., 1995). The signals from the most stable 5-seconds out of the 10-seconds lasting period were used.
**B**	**Reference posture**
Posture	Subjects stood comfortably upright, heels, buttocks and upper back touching the wall, arms hanging and head facing straight ahead. The signals from a stable 1-second period of the 5-second posture were used.

Appendix 2

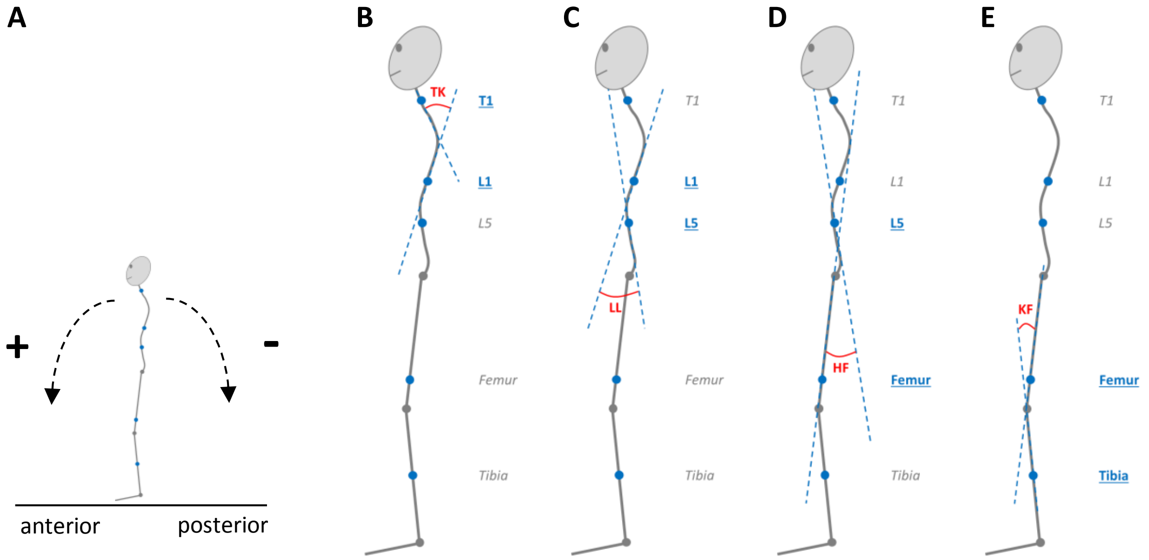

Appendix 2. A: An anterior inclination of the position sensors generates positive values. A posterior inclination of the position sensors generates negative values. **B-E:** The anteroposterior tangent lines of the position sensors placed over the processus spinossi were used for the four predefined angle calculations according to the cobb method (Takács et al., 2018). **B:** Thoracic kyphosis = T1-L1. **C:** Lumbar lordosis = L1-L5. **D:** Hip flexion = L5-Femur. **E:** Knee flexion = |Femur-Tibia|. Prior to the calculations the algebraic signs of the sensors placed on femur and tibia were corrected, means positive values were changed to negative and negative to positive, as the sensors were placed on the anterior body side and therefore mirror-inverted to the sensors placed over the spine.

References

Biering-Sørensen, F. (1984). Physical measurements as risk indicators for low-back trouble over a one-year period. *Spine, 9*(2), 106-119. https://doi.org/10.1097/00007632-198403000-00002

Mathiassen, S. E., Winkel, J., & Hägg, G. M. (1995). Normalization of surface EMG amplitude from the upper trapezius muscle in ergonomic studies - A review. *Journal of Electromyography and Kinesiology, 5*(4), 197-226. https://doi.org/10.1016/1050-6411(94)00014-x

Takács, M., Orlovits, Z., Jáger, B., & Kiss, R. M. (2018). Comparison of spinal curvature parameters as determined by the ZEBRIS spine examination method and the Cobb method in children with scoliosis. *PloS One, 13*(7), e0200245. https://doi.org/10.1371/journal.pone.0200245
